# Sequence mutations of the substrate binding pocket of stem cell factor and multidrug resistance protein ABCG2 in renal cell cancer: A possible link to treatment resistance

**DOI:** 10.3892/or.2013.2324

**Published:** 2013-03-05

**Authors:** INKA ZOERNIG, CLAUDIA ZIEGELMEIER, BERND LAHRMANN, NIELS GRABE, DIRK JÄGER, NIELS HALAMA

**Affiliations:** 1 Department of Medical Oncology, National Center for Tumor Diseases, University of Heidelberg, D-69120 Heidelberg, Germany; 2 Tissue Imaging and Analysis Center (TIGA) and National Center for Tumor Diseases, D-69120 Heidelberg, Germany

**Keywords:** ABCG2, multidrug transporter, treatment resistance

## Abstract

ABCG2 is a multidrug cellular transport protein that is associated with resistance to certain treatments in patients, particularly anticancer treatment. The tumor-protective properties of ABCG2 expression are reported to be a feature of a subset of stem cell-like tumor cells. While protection against chemotherapy has been well analyzed, the role of ABCG2 in the treatment with tyrosine kinase inhibitors is only partially understood. Tyrosine kinase inhibitors are currently the main treatment option in irresectable renal cell carcinomas. To investigate possible underlying sequence variations in the ABCG2 gene with relevance to the functional properties of the protein, 36 patient samples were analyzed. Using sequence analysis and single-nucleotide polymorphism databases, sequence variations in the highly conserved domains of the binding pocket of ABCG2 were analyzed. The resulting variations were used for computational protein prediction algorithms to identify conformational alterations. A relevant shift from A to G at position 1376 (resulting in Y→C at 459 aa) was identified and found to be present in 8.3% of the patients. These patients are currently in follow-up after resection, thus, further analysis will reveal whether this mutation has relevance to treatment efficacy.

## Introduction

During the last decades, the development and clinical use of anticancer drugs has become a regularity and therefore an important way of controlling malignancies. However, tumors can develop drug resistance to different therapeutic drugs, making available chemotherapeutic agents ineffective in the course of the disease. Among the proteins involved in anticancer drug resistance are the ATP-binding cassette (ABC) transporters (1). ABC transporters constitute the largest superfamily of human cellular transporters. There are currently 48 members classified in seven subfamilies termed A to G. These transporters have the ability to actively transfer a multitude of structurally dissimilar endogenous and exogenous substrates and their metabolites across cell membranes (2). ABCG2 (also known as the breast cancer resistance protein/mitoxantrone-resistance/ABC protein) consists of 655 amino acids, one transmembrane domain with six putative transmembrane segments, and a single ATP-binding site. Deduced from its structure, ABCG2 is a ‘semi-transporter’ (3–5) similar to other members of the ABCG subfamily including the *Drosophila* White protein ortholog (6). Overexpression of ABCG2 has been shown to confer resistance to a variety of chemotherapeutic agents. Affected drugs are anthracenedione mitoxantrone (7,8); the camptothecin derivatives, topotecan (9,10) and SN-38 (11); the anthracycline doxorubicin (12); and the antifolate methotrexate (13–15). Mutations in the ABCG2 gene have been associated with high-level anticancer drug resistance (16). Furthermore, the effect of tyrosine kinase inhibitors on ABCG2 has been reported (17). Pharmacogenetic studies showed influences on pharmacokinetics of tyrosine kinase inhibitors through mutations in the ABCG2 coding sequence (18). The use of the tyrosine kinase inhibitor imatinib has been shown to overcome cancer drug resistance via ABCG2, whereas the efflux function is inhibited by tyrosine kinase inhibitors. This functionality was shown to be mediated through an interaction with ABCG2 at the substrate binding site (19). Recent structural analyses identified transmembrane domain 3 (around amino acid position 482) as the potential substrate-binding pocket (20). In medical oncology, tyrosine kinase inhibitors have become important drugs in the treatment of renal cancer and therefore predictive markers for treatment efficacy are of interest. To date, no data on mutations in the potential substrate binding pocket of ABCG2 in tumor tissue are available. Therefore, our aim was to investigate mutations in the coding region for the transmembrane domain 3 and the surrounding domains of ABCG2 in numerous renal cancer samples. Thus, we directly sequenced the corresponding cDNA taken from 36 renal cancer tumor samples.

## Materials and methods

### Patient samples

Tissue samples were obtained from 36 renal cell carcinoma patients who underwent elective surgery in the University Hospital Heidelberg, Germany, after giving their informed consent and following the ethics approval of the respective committees. The sample of tumor tissue (200–500 mg) was obtained from a central part of the respective carcinoma. Only non-necrotic tissue was collected. All samples were snap frozen in liquid nitrogen and stored at −80°C until further examination. Approximately 20–200 mg of tumor sample was subjected to DNA and RNA extraction.

### Extraction of RNA

For RNA extraction, tissue samples from renal cell carcinoma patients were subjected to homogenisation and lysis using the Qiagen RNA kit (Qiagen, Hilden, Germany). Isolated RNA was measured by spectrophotometry.

### Synthesis of cDNA from RNA

Reverse transcription was performed with the Superscript III reverse transcriptase (Invitrogen) and random hexamer primers (Applied Biosystems). The reaction mix was incubated for 5 min at 25°C, 60 min at 50°C and finally at 70°C for 15 min to inactivate the reverse transcriptase.

### Amplification of ABCG2 sequence fragments and detection of ABCG2 mutations by sequencing

The following set of primers was used to amplify a fragment of 690 bp. Forward primer sequence was 5′-tggagattccactgctgtggca and reverse primer sequence was 5′-tgacctgctgctatggccagtg, annealing temperature was 60.5°C. Reaction volume was 25 μl, with 17.5 μl RNAse-free water, 2.5 μl 10X PCR buffer, 0.75 μl MgCl_2_ 50 mM, 2 μl dNTP mix 10 mM, 0.5 μl of each primer (10 μM) and 0.25 μl of Taq polymerase. cDNA (1.0 μl) was added. Thirty-five cycles were performed on an MJ Research PCR Engine with an initial denaturation of 10 min. A cycle consisted of 1 min denaturation of 95°C, 60.5°C annealing for 1 min and 1 min at 72° for extension. Sequencing reactions were then performed on the PCR products with the respective sequencing primer and the 3′Big Dye Terminator Cycle Sequencing Ready Reaction kit (ABI, Weiterstadt, Germany) according to the manufacturer's instructions.

### Identification of possible functional single-nucleotide polymorphisms (SNPs) affecting the region of interest

Annotated SNPs from dbSNP (http://www.ncbi.nlm.nih.gov/entrez/query.fcgi?db=snp) and the HapMap project (http://www.hapmap.org/) in the corresponding genomic region of the ABCG2 gene were included in the analysis. The following coding non-synonymous SNPs were analyzed (21,22): rs41282401, rs9282571, rs3201997, rs3116448, rs2231142, rs1061018 and rs1061017. Coding synonymous SNPs were: rs12721640, rs3116439 and rs2231139.

### Computational protein secondary structure prediction

To evaluate the impact of an amino acid change we used the JPRED software (http://www.compbio.dundee.ac.uk/www-jpred/) (23). This software predicts the secondary structure using a neural network called Jnet. The prediction is the definition of each residue into either α helix, β sheet or random coil secondary structures (24,25). Predictions were generated for the unaltered protein sequence and for the corresponding mutated protein. Predictions were then compared by visual inspection.

Additionally, a secondary structure prediction was performed with the ESyPred3D program (26), accessible through http://www.fundp.ac.be/sciences/biologie/urbm/bioinfo/esypred/. A secondary structure could be computed for the unaltered protein, but no secondary structure prediction was possible for the mutated protein.

## Results

### Comparative proteomics analysis

**Figure 1:**
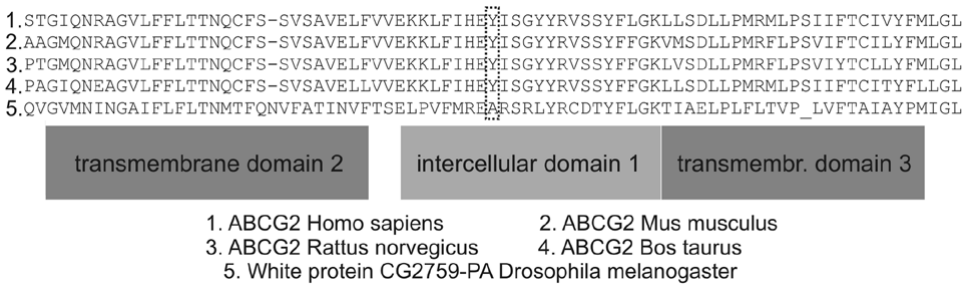
Alignment of ABCG2 protein sequences (in *Homo sapiens*, *Mus musculus*, *Rattus norvegicus*, *Bos taurus* and the White-protein of *Drosophila* melanogaster) corresponding domains and conservation scores for different positions (18,20).

To further elucidate the structural organization of the domains in the region of interest, a computational analysis was performed. BLASTp searches and a PSI-BLAST against the whole protein, the ABC_Transporter_2 domain (PS50893) and the ABC2-membrane domain (PF01061) revealed a set of highly conserved residues. In Fig. 1, the position-specific conservation scores and a subset of aligned protein sequences are shown.

### Sequence analysis

Detailed examination of the raw sequences and the automated sequences revealed a heterozygous shift from A to G at position 1376 (Y→C at 459 aa, ICD1a, intracellular domain 1a, a position which is highly conserved) in 3 patients. Of note, none of the known SNPs was detected. Sequencing was repeated when the acquired sequence data was ambiguous.

### Computational protein folding prediction

**Figure 2:**
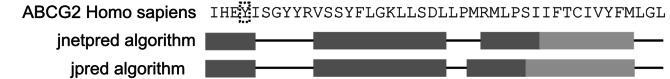
Predictions generated with JPRED for the wild-type sequence; ‘query’ sequence is the human ABCG2 protein sequence; dotted lines highlight position 459; bottom lines show schematic predictions (dark grey boxes represent helical secondary structure and light grey boxes represent extended secondary structures).

**Figure 3:**
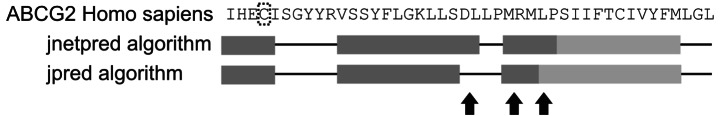
Predictions generated with JPRED for the mutated sequence; ‘query’ sequence is the human ABCG2 protein sequence; dotted lines highlight position 459; bottom lines show schematic predictions (dark grey boxes representing helical secondary structure and light grey boxes represent extended secondary structures; short black arrows indicate regions of altered folding prediction downstream of the mutation).

The prediction with JPRED showed a subsequent change in the downstream folding of the protein induced by the mutation. The structures of the intercellular domain 1b and 1c and of the transmembrane domain 3 (TM3) were changed (Figs. 2 and 3).

### Association with clinical data

Tumor samples with mutations led to evaluation of the clinical course of the corresponding patient. All 3 patients had surgical resection of the tumor and are in regular follow-up. To date, no active tumor disease has been found in the 3 patients. Therefore, no clinical data on treatment response were available for these patients.

## Discussion

Cancer drug resistance is a problem usually encountered in prolonged chemotherapy. A significant proportion of patients has no primary response to certain chemotherapeutic agents. Understanding the mechanisms behind this drug resistance on the molecular level can lead to improved therapeutic approaches. In the case of renal carcinoma, the introduction of tyrosine kinase inhibitors marked the beginning of a new therapeutic era. Thus far, mechanisms for tumor drug resistance against tyrosine kinase inhibitors in renal cancer cells have not been identified.

The ABCG2 gene was identified as a potent ‘de-toxification’ transporter for tyrosine kinase inhibitors in cancer cells. In renal cancer cell lines, aberrant promoter methylation of the ABCG2 gene was shown (27) and multiple SNPs have been reported in patients with renal cancer (28), but no direct analyis of tumor tissue samples or a direct association with resistance to tyrosine kinase inhibitors has been reported. Herein we report an analysis of the corresponding cDNA sequence of the probable substrate binding pocket of ABCG2 by direct sequencing. It has been hypothesized that residue R482 in the transmembrane domain 3 (TM3) is likely to interact with substrates based on the effect of R482G/T mutations (29). The aforementioned mutations generated gain-of-function mutants, resulting in resistance to a wider range of substrates than the wild-type transporter.

Further mutagenesis studies showed that replacement of arginine with virtually any residue that was not positively charged led to a similar gain-of-function or change-of-function (30). Based on these findings, it was speculated that R482 must be part of the substrate-binding pocket in the structure (20). Our data shows a mutation resulting in Y459C in the intracellular domain 1a (ICD1a). This position was identified as highly conserved (20) and showed heterozygous mutations from A to G at the nucleotide position 1376 in 3 out of 36 tumor samples. Based on the actual models (20), the apparent flexibility of the ICD1 may play a role in transmitting conformational changes from the nucleotide binding domain to the transmembrane domain or vice versa. The prediction of JPRED regarding secondary folding showed an alteration in the adjacent helical structure supporting the hypothesis of a structural change associated with this mutation.

Two mutation-bearing tumor samples were clear cell renal carcinomas, one sample included a chromophobe renal cell carcinoma, therefore, association of the mutation with a certain histological tumor type was not possible.

In future studies we will investigate tumor tissue from renal cancer patients who receive treatment with tyrosine kinase inhibitors to determine whether this mutation is associated with increased tumor drug resistance or good response to therapy with tyrosine kinase inhibitors.

The estimated rate of 8.3% among all renal cancer patients makes this mutation particularly attractive with regard to a possible pre-estimate of (non-surgical) therapeutic efficacy. This is the first report on the sequence analysis of the substrate binding pocket ABCG2 from tumor tissue of renal cell carcinoma.
